# Neurocognitive Impact of Exposure to Cannabis Concentrates and Cannabinoids Including Vaping in Children and Adolescents: A Systematic Review

**DOI:** 10.7759/cureus.52362

**Published:** 2024-01-16

**Authors:** Michell S Saavedra, Priyanka Thota, Tariladei S Peresuodei, Abhishek Gill, Chijioke Orji, Maiss Reghefaoui, Safeera Khan

**Affiliations:** 1 Medicine, University of Cuenca, Cuenca, ECU; 2 Pediatrics, California Institute of Behavioral Neurosciences and Psychology, Fairfield, USA; 3 Medicine, Siddhartha Medical College, Vijayawada, IND; 4 Internal Medicine, California Institute of Behavioral Neurosciences and Psychology, Fairfield, USA; 5 Trauma and Orthopedics, Betsi Cadwaladr University Health Board, Wrexham, GBR; 6 Trauma and Orthopedics, California Institute of Behavioral Neurosciences and Psychology, Fairfield, USA; 7 Internal Medicine, University of Debrecen, Debrecen, HUN

**Keywords:** neurocognitive changes, vaping, cannabinoid, cannabis, adolescent, pediatric population

## Abstract

During adolescence, significant changes unfold in the brain's maturation process. The density of white matter increases, accompanied by the pruning back of gray matter. This critical and vulnerable period becomes especially noteworthy in the context of drug use, as adolescents are extensively exposed to substances such as tobacco, alcohol, and cannabis. The concern is heightened now that cannabis has been legalized for recreational use in many places, leading to increased exposure levels. Additionally, knowledge about the impact of cannabis on neurocognitive development during this stage is limited. This knowledge gap compounds the issue, making it even more concerning. Therefore, a systematic review was carried out based on the 2020 Preferred Reporting Items for Systematic Reviews and Meta-Analyses (PRISMA) guidelines, using medical databases such as PubMed, PubMed Central (PMC), Medline, Cochrane Library, Internet Archive Scholar, and Embase-Elsevier for relevant medical literature. The identified articles were reviewed, eligibility criteria were applied, and 19 research articles were identified. The final papers explored the correlation between children's and adolescents' exposure to cannabis-containing compounds and subsequent changes in the central nervous system (CNS). Findings revealed a considerable impact, ranging from transient alterations in mood to permanent cognitive function and sensory processing changes, affecting the deterioration of the quality of life of these individuals in adulthood. Presently, most studies were conducted on animals, and the few studies on humans have considerable limitations, such as the type of study, age of the population, and small samples, among others. For this reason, it is essential for the scientific community and public health organizations, in general, to conduct more studies that demonstrate the true neurobiological impact of this drug and its accessibility to young people and, based on the results, consider its legalization or propose regulations for its use and commercialization.

## Introduction and background

Since the early 2000s, cannabis has been legalized in many countries for medical and recreational purposes. In the United States (as of January 2023), 45 out of the 51 states have legalized medical cannabis, with 22 also approving it for recreational use. Canada fully legalized it in 2018, while in South America, Europe, and Australia, legalization has been limited to medical purposes, with a few exceptions [1].

In 2020, cannabis emerged as the most-consumed drug globally, attracting 209 million users. The rate of adolescent consumption shows an annual increase [1]. Excluding tobacco and alcohol, cannabis stands out as the most prevalent drug among young people aged 15-24, according to the United Nations. On average, around 9% of cannabis users develop use disorders, and this figure rises to over 16% for those initiating use during adolescence [2].

Adolescents are particularly susceptible to the influence of drugs due to the fully developed limbic system combined with the ongoing development of their decision-making cortical areas. The interplay of innate curiosity, a natural instinct to explore new experiences, and a potential misperception of its safety of use [1], coupled with the growing availability and diverse consumption methods like oral consumption (edibles) and vaporization, alongside the progressive acceptance through legal changes worldwide, poses a significant threat to global public health [3].

A number of previous studies have shown the adverse effects of chronic cannabis consumption on the pulmonary, respiratory, and cardiovascular systems. While further research is necessary, there are indications of cannabis impact on the central nervous system (CNS), particularly when consumption begins at an early age (<16 years) [1,4]. This early onset has been associated with mental health disorders, including personality disorders, depression, and suicidality. Moreover, there is evidence that acute consumption, even infrequently, can lead to negative mood states such as anxiety, mental confusion, tension, memory impairment, instability, and paranoia [2].

Adolescence, typically spanning from approximately 10 to 19 years old, is marked by a series of developmental changes, including brain maturation. This phase exhibits sensitive periods of development that vary across different brain regions. It is crucial to facilitate the development of cognitive skills, such as enhancements in intelligence quotients, working memory, problem-solving, and behavioral skills, including risk-taking, sensation-seeking, and socialization. This stage represents a sensitive period during which the brain is particularly susceptible to substance use [5]. The outcomes of such substance use play a pivotal role in shaping human behavior, as they result from the process of "biological embedding," influenced by both social and environmental conditions [2].

The consumption of cannabis during adolescence is a matter of particular concern, as it signifies shifts in social and political perceptions and poses scientific, medical, and economic challenges. The ongoing wave of cannabis legalization must be accompanied by new responsibilities for educating the community about the known dangers and potential risks associated with both recreational and medicinal use [2,6]. This emphasizes the critical need for more scientific evidence to strengthen the understanding of the potential effects of cannabis on neurocognitive development during childhood and adolescence, as only a limited number of studies have been conducted in humans [7]. Results often vary, primarily due to the constraints of small sample sizes [8].

Given this lack of concrete results and the possibility of future negative impacts on society, such as the correlation between consumption and lower quality of life [9], it is essential to inquire more about this topic. For this reason, the following question arises: Does the pediatric population exposed to cannabis and cannabinoid-containing chemicals (including vaping) exhibit neurocognitive changes compared to those not exposed?

## Review

Methodology

The systematic review was conducted based on the Preferred Reporting Items for Systematic Reviews and Meta-Analyses (PRISMA) 2020 guidelines.

Search Strategies

We researched databases like PubMed, PubMed Central (PMC), Medline, Cochrane Library, Internet Archive Scholar, and Embase-Elsevier. We use various combinations of our keyword concepts, pediatric, cannabis, cannabinoid, vaping, and neurocognitive, to search all databases. In PubMed, however, along with these keywords, the following strategy was developed and used to search relevant literature in PubMed's Medical Subject Headings (MeSH) database: ((Cannabis OR Cannabinoid OR Marijuana OR ("Cannabis/adverse effects" Majr OR "Cannabis/drug effects" Majr OR "Cannabis/growth and development" Majr OR "Cannabis/toxicity" Majr)) OR (Vaping OR THC Vaping OR ("Vaping/adverse effects" Mesh OR "Vaping/cerebrospinal fluid" Mesh OR "Vaping/pathology" Mesh))) AND (Neurocognitive changes OR "Neurocognitive Disorders/etiology" Majr) AND (Pediatric population or children or adolescent OR "Pediatrics/classification" Majr). All the search strategies, the databases used, and the identified number of papers for each database are shown in Table 1.

**Table 1:** Keywords/strategy used and number of identified papers. MeSH: Medical Subject Headings

No.	Keywords/search strategy	Database used	Number of papers identified
1	Marijuana AND Pediatric	Cochrane Library	19
2	((Cannabis OR Cannabinoid OR Marijuana OR ("Cannabis/adverse effects" Majr OR "Cannabis/drug effects" Majr OR "Cannabis/growth and development" Majr OR "Cannabis/toxicity" Majr)) OR (Vaping OR THC Vaping OR ("Vaping/adverse effects" Mesh OR "Vaping/cerebrospinal fluid" Mesh OR "Vaping/pathology" Mesh))) AND (Neurocognitive changes OR "Neurocognitive Disorders/etiology" Majr) AND (Pediatric population or children or adolescent OR "Pediatrics/classification" Majr)	PubMed MeSH database	94
3	Cannabis AND Neurocognitive AND Children OR adolescents	PubMed	127
4	Cannabinoid AND Neurocognitive AND Children OR Adolescents	PubMed	37
5	Children AND Adolescents AND Exposure to cannabis	PubMed	282
6	Pediatric exposure AND Cannabis OR Cannabinoids	PubMed	297
7	Neurocognitive changes in pediatric population after exposure to cannabis, cannabinoids, vaping	Google Scholar	134
8	Cannabis, cannabinoids, or vaping exposure in children and adolescents	Embase	111
9	Postnatal cannabis exposure in children and adolescents	Embase	431
Total number of research papers identified			1532
Number of articles after removing duplicates			824

Eligibility Criteria

All the inclusion and exclusion criteria are mentioned in Table 2.

**Table 2:** Inclusion and exclusion criteria. THC: tetrahydrocannabinol

No.	Inclusion criteria	Exclusion criteria
1	Papers written and published in English and Spanish and those able to translate to these languages	Gray literature: encyclopedia, conference abstracts, conference information, discussion, editorials, practice guidelines, short communications, and proposal papers
2	Papers from the past five years	Exposure to other recreational drugs apart from cannabis and cannabinoids, including THC vaping
3	Papers focusing on neurocognitive changes in children and adolescents after exposure to compounds containing cannabis, cannabinoids, or THC vaping	Studies performed on animals
4	Papers focusing on children and adolescent groups	Articles focusing on exposure in children or adolescents with any other baseline condition
5	Article type: review articles, research articles, and book chapters	No full text is available
6	Papers involving human subjects	
7	Papers include full texts	

Selection Process

All articles were thoroughly checked, and duplicate reports were removed. Each paper was screened through titles and abstracts. In case of a conflict about eligibility, concerns were discussed with all co-authors, and by mutual consensus, the preselected articles were chosen. The final number of shortlisted articles was determined by evaluating the same ones using the full text and applying inclusion and exclusion criteria (Table 2).

Quality Appraisal of the Studies

The quality of the shortlisted articles was checked using relevant quality assessment tools. In case of any conflict, the problems were discussed with all the co-authors, and the final decision to include the article was made by mutual consensus.

The Cochrane bias assessment tool was used to evaluate a randomized controlled trial. The Scale for the Assessment of Narrative Review Articles (SANRA) checklist was utilized to assess the quality of narrative reviews, while observational studies were considered using the Newcastle-Ottawa tool. A MeaSurement Tool to Assess systematic Reviews (AMSTAR) tool was used for evaluating systematic reviews. Only studies that satisfied the quality appraisal were included in the systematic review. Table 3, Table 4, Table 5, and Table 6 comply with the quality appraisal of only those studies included in the review.

**Table 3:** Quality appraisal using the Cochrane bias assessment tool for clinical trials.

Clinical trials	The bias of the randomization process	Effect of assignment on intervention	Effect of adhering to an intervention	Bias due to missing outcome data	Bias in the measurement of outcome	Bias in the selection of reported results
Cuttler et al. [10]	Low risk	Low risk	Low risk	Low risk	Medium risk	Low risk

**Table 4:** Quality appraisal using SANRA. SANRA: Scale for the Assessment of Narrative Review Articles

Reviews	Justification of the article's importance for readership	Formulation of specific aims or questions in a clear and concise manner	Description of literature search	Referencing	Scientific reasoning	Appropriate presentation of data
Scheyer et al. [2]	2	2	1	2	2	2
Morie and Potenza [9]	2	0	1	2	2	2
Bara et al. [11]	2	1	2	2	2	2
Hammond et al. [12]	2	1	1	2	2	2

**Table 5:** Quality appraisal using the Newcastle-Ottawa tool.

Study	Selection	Comparability	Outcome
Ho et al. [5]	++++	++	+++
Lichenstein et al. [13]	+++	+	+++
Albaugh et al. [7]	++++	++	+++
Wang et al. [14]	+++	++	+++
Frolli et al. [15]	++++	++	++
Meier et al. [16]	++++	+	++

**Table 6:** Quality appraisal using AMSTAR. AMSTAR: A MeaSurement Tool to Assess systematic Reviews

AMSTAR criteria	Pintori et al. [1]	Hammond et al. [3]	Lichenstein et al. [4]	Allick et al. [17]	Duperrouzel et al. [8]	Figueiredo et al. [18]	Campeny et al. [19]	Jacobus et al. [20]
1. Was an a priori design provided?	Yes	Yes	No	Yes	No	Yes	No	No
2. Were there duplicate study selection and data extraction?	No	Yes	No	Yes	No	Yes	Yes	No
3. Was a comprehensive literature search performed?	Yes	Yes	Yes	Yes	Yes	Yes	Yes	Yes
4. Was the status of publication used as an inclusion criterion?	Yes	Yes	No	No	No	No	No	No
5. Was a list of studies provided?	Yes	Yes	Yes	Yes	Yes	Yes	Yes	Yes
6. Were the characteristics of the studies included in the analysis provided?	Yes	Yes	Yes	Yes	Yes	Yes	Yes	Yes
7. Was the scientific quality of the included studies assessed and documented?	No	Yes	No	Yes	Yes	Yes	Yes	Yes
8. Was the scientific quality of the included studies used appropriately in formulating conclusions?	Yes	Yes	No	Yes	Yes	Yes	Yes	Yes
9. Were the methods used to combine the findings of studies appropriate?	Yes	Yes	Yes	Yes	Yes	Yes	Yes	Yes
10. Was the likelihood of publication bias assessed?	Yes	Yes	Yes	Yes	No	Yes	Yes	Yes
11. Was the conflict of interest stated?	Yes	Yes	Yes	Yes	Yes	Yes	Yes	No
Total score (out of 11)	9	11	6	10	7	10	9	7
Overall methodology quality (L=low, M=moderate, H=high)	H	H	M	H	M	H	H	M

Data Collection Process

The primary results were evaluated after extracting the final articles for the systematic review. MS extracted the data independently, and with the unanimous participation of all authors, the data extraction was completed.

Results

Study Identification and Selection

**Figure 1:**
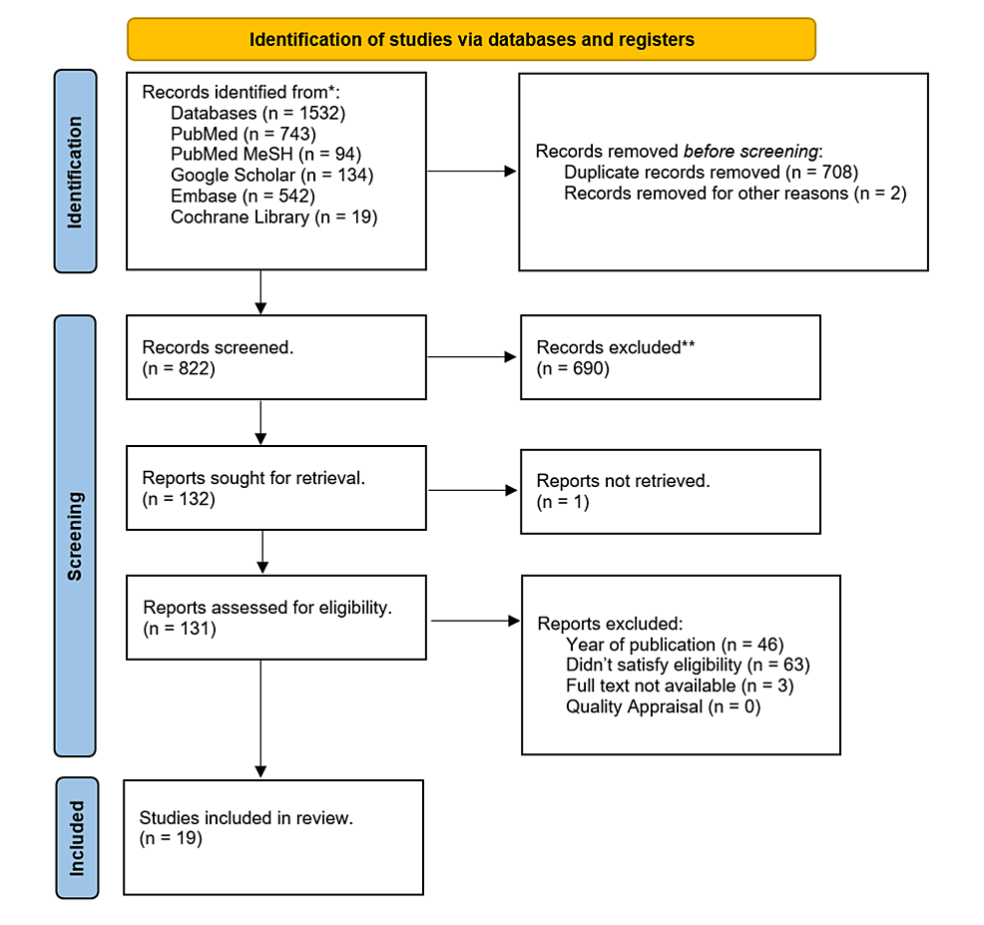
PRISMA flowchart outlining the article selection process. PRISMA: Preferred Reporting Items for Systematic Reviews and Meta-Analyses

We gathered a total of 1532 relevant studies using all the databases. Of them, 708 duplicate studies were removed, and two were released for other reasons before the screening. After screening the remaining studies based on titles, abstracts, retrieving full text, inclusion and exclusion criteria, and full-text articles, 19 articles were shortlisted. These shortlisted full-text articles were used for quality appraisal and final review; eight were systematic reviews, six were nonrandomized clinical trials, four were narrative reviews, and one was a randomized clinical trial. Figure 1 of the PRISMA flowchart shows the selection process for finalized studies.

Detailed analyses of outcomes measured in the studies are shown in Table 7.

**Table 7:** Summary of the included studies. THC: tetrahydrocannabinol; ATR: anterior thalamic radiations; FA: fractional anisotropy; CBD: cannabidiol; CU: cannabis users; fMRI: functional magnetic resonance imaging; rmPFC: rostral medial prefrontal cortex; mPFC: medial prefrontal cortex; TD: typically developing; CUD: cannabis use disorder; GMV: gray matter volume

No.	Authors and year of publication	Type of the study	Purpose of the research and biomarker studied	Number of participants	Results	Conclusions
1	Cuttler et al. [10]	Randomized clinical trial	Investigate the short-term memory and decision-making impact of high-dose cannabis flower (20% THC) and concentrates (60%)	80	The use of cannabis has been found to significantly impair free recall and increase the likelihood of false memories for related and unrelated phrases but not for essential cues. It is important to conduct further research to determine whether cannabis concentrates may exacerbate or mitigate the potentially negative impacts of acute cannabis use on cognitive and physical health	This study investigates the immediate effects of cannabis on various aspects of cognitive function, such as temporal order memory, resistance to biases, over-/under-confidence, and consistency in risk perception. Even though high-potency cannabis was used, no noticeable changes were observed in the results
2	Ho et al. [5]	First: prospective longitudinal study; second: cohort study	Cognitive development and susceptibility to schizophrenia in adolescents who use recreational marijuana	First (Iowa) sample: 211; second (birth cohort) sample: 3463	Iowa sample: marijuana use was significantly associated with lower cognitive performance in sustained attention, visuospatial working memory, and executive sequencing. In the birth cohort, individuals who engaged in more recreational and regular cannabis use demonstrated decreased focused attention	Cannabis use during teenage years can interfere with normal development and increase the risk of familial schizophrenia. It is crucial to restrict teen access to cannabis
3	Lichenstein et al. [13]	Observational study	Examined the relationship between adolescent and emerging adult cannabis usage and the white matter architecture of the cingulum and ATR	158	Compared to little and extensive teenage usage, moderate cannabis use during adolescence (12-19) was related to increased cingulate FA and ATR. Reduced positive change in cingulate FA between ages 20 and 22 was found to be associated with long-term moderate and high cannabis use (12-21 years)	Cannabis exposure may have an impact on the timing of the transition to adulthood, which could potentially affect how individuals function in their future lives
4	Albaugh et al. [7]	Retrospective cohort study	The impact of cannabis use during adolescence on neurodevelopment	799	There is no correlation between the thickness of the left and right prefrontal cortices. At a five-year follow-up, no statistically significant correlation existed between lifetime cannabis use and baseline cortical thickness, indicating that the observed neuroanatomical differences may not be related to cannabis use	The findings imply that cannabis usage during adolescence is linked to altered neurodevelopment, especially in mid-to-late-adolescent cortices that are rich in cannabinoid receptor 1 and going through significant changes in thickness due to aging
5	Wang et al. [14]	Longitudinal study	Alterations in cerebellar thickness brought on by excessive cannabis use	44	At first, regular cannabis use was associated with lobule VI and Crus I changes	Cannabis usage alters the cerebellum structure and tracking changes. These can serve as biomarkers that may assist in creating treatment tools
6	Frolli et al. [15]	Cross-sectional study	Cognitive development in adolescent cannabis users	300	According to statistical analysis, people who regularly use cannabis perform significantly worse on working memory and processing speed tasks than non-users and occasional users	Future research could confirm the number of neurocognitive abnormalities through controlled follow-up evaluations and analysis of neurofunctional data
7	Meier et al. [16]	Retrospective cohort	Boys who have used cannabis recently and over time may experience internalizing problems from adolescence to young adulthood	-	The findings demonstrated a correlation between recent increases in cannabis usage and cumulative past years of weekly cannabis use with increases in depressive symptoms and anxiety/depressive disorders. Boys who used cannabis every week for extended periods displayed increased internalizing issues, indicating the significance of stopping chronic weekly cannabis usage	Chronic marijuana use has been linked to an increase in internalizing problems. Internalizing problems and previous cumulative cannabis use were associated to a small extent
8	Scheyer et al. [2]	Narrative review	Lasting cognitive impairments and underlying mechanisms in adolescent cannabis users	-	Preclinical research demonstrates that cannabis impacts behavior and neurodevelopmental processes during windows of vulnerability (such as adolescence). It alters dendritic structures and their synaptic activities (including those mediated by endogenous cannabinoids and neural circuits) in a long-lasting manner	An understanding of how cannabis exposure might work to increase neuropsychiatric risk and how cannabinoids may interact with environmental factors (such as acute or chronic stress) during adolescence is urgently needed to raise awareness of disease risk
9	Morie and Potenza [9]	Systematic review	Relationship between cannabis use and neural foundations of reward processing, inhibitory control, and working memory	-	Chronic cannabis usage, especially high THC strains, is linked to changes in brain activity and actions involved in processing rewards, working memory, and inhibitory control. The possible effect of CBD in these fields seems minimal or nonexistent	There are few imaging investigations of emotional control in cannabis use disorders. One study found that when those who use cannabis are compared to those who don't, there is less activation in the precentral and mid-cingulate regions of the bilateral frontal lobes during the emotional reappraisal of negative affect. Future studies are required to understand better how THC and CBD affect emotion modulation
10	Bara et al. [11]	Narrative review	Synaptic reprogramming of the developing brain in cannabis users	-	Exposure during the prenatal and teenage ages alters different neurobiological systems' trajectories. The evidence also shows that despite THC exposure being restricted to the early stages of brain development, disturbance of synaptic plasticity is a typical feature of the adult brain	Systematic research is essential to pinpoint risk factors for those who might develop psychopathologies and to maximize early intervention for prevention
11	Hammond et al. [12]	Narrative review	Changes in use patterns, comorbidity, and health correlates among US adolescent cannabis users	-	According to the most recent research, cannabis use among adolescents is linked to several detrimental life outcomes, such as cognitive impairments, a rise in the prevalence of psychotic, mood, and addiction disorders, and a worsening course	Federal and state governments should use the public health framework to consider any potential negative consequences of changing marijuana laws on children's health
12	Pintori et al. [1]	Systematic review	Adolescent exposure to THC, CBD, and its effect	-	As a result of the widespread availability of CBD and THC in various forms, people, including young people, are consuming CBD because it is thought to be "safe." The adverse effects of THC are evaluated in a large body of literature. However, nothing is known about the long-term effects of CBD consumption, particularly in adolescents. CBD indicates improvement in anxiety, antipsychotic, neuroprotective, or neuroinflammatory results	The information on the rewarding and addictive properties of CBD and how its use throughout adolescence may influence the emergence of substance use disorders is severely lacking. To fully comprehend the impact of CBD therapy and its potential therapeutic effects, particularly in adolescence, a better preclinical and clinical characterization of CBD is required
13	Hammond et al. [3]	Meta-analysis	Alterations in executive control, social cognition/emotion processing, and reward processing in cannabis using youth (fMRI studies)	-	Teenage CU showed increased rmPFC activation and decreased dorsal mPFC activation during executive control and social cognition/emotion processing, compared to non-using TD youth	In cortical and subcortical brain regions, there are changes in neuronal response between CU and TD kids during executive control, emotion processing, and reward processing. These differences depend on sex, the degree of CUD, psychiatric comorbidity, period of abstinence, and whether standard predisposition variables can explain abnormal brain function in CU kids
14	Lichenstein et al. [4]	Systematic review	Research has been conducted on the structural and functional changes in the brain due to cannabis use during adolescence and emerging adulthood	9441	There is preliminary evidence that adolescent cannabis users have functional and structural changes in the frontoparietal, frontolimbic, frontostriatal, and cerebellar regions	To reconcile conflicting findings and evaluate potential modifiers of cannabis' effects on the developing brain, further thorough research is necessary
15	Allick et al. [17]	Systematic review and meta-analysis	There are differences in cortical gray matter volume related to age and sex among adolescent cannabis users	-	The investigations failed to detect any areas with a discernible difference in GMV between CU and TD children. Age and sex had different effects on cortical GMV differences in young CU vs. TD, according to meta-regressions	According to these results, any GMV differences between CU and TD youth may be minor and depend on a young person's age, cumulative cannabis exposure, and sex
16	Duperrouzel et al. [8]	Systematic review and meta-analysis	The negative impact of using cannabis on the cognitive abilities of the brain	-	Healthy regular cannabis users have impaired brain function compared to non-users and worse neurocognitive functioning across multiple neurocognitive domains	It is necessary to conduct further research, especially large-scale longitudinal studies, to determine the key moments or usage patterns more likely to have detrimental effects
17	Figueiredo et al. [18]	Systematic review and meta-analysis	A neurocognitive consequence of chronic cannabis use	-	Chronic cannabis use has been linked cross-sectionally to deficits in six areas of neurocognition, including memory, cognitive impulsivity, flexibility, and attention	Future research should focus on characteristics of cannabis use in carefully selected subjects
18	Campeny et al. [19]	Systematic review	Cannabis use-related health harms	-	Cannabis use is linked to psychosis, affective disorders, anxiety, sleep disorders, cognitive decline, unfavorable respiratory events, cancer, cardiovascular outcomes, and gastrointestinal diseases, according to the evidence. Cannabis use increases the chances of car accidents, suicidal thoughts, and domestic violence	Cannabis use increases the likelihood of several medical disorders and adverse social effects. The evidence needed to identify these effects from the public health angle is still lacking regarding their dose dependency
19	Jacobus et al. [20]	Systematic review	Cannabis and the developing brain	-	Recent cannabis usage, frequency, and age at which cannabis use started are probably essential factors in predicting outcomes with worse brain health. There is some proof that pre-existing variations in brain design could affect vulnerability and differences in results	It will be possible to separate how cannabis usage and pre- and post-exposure variations play a role in different outcomes among youth who use cannabis through ongoing large-scale prospective studies of young people

Discussion

Cannabis

This plant belongs to the Cannabaceae family and is native to Central Asia. First, it was used as a medicinal herb with therapeutic purposes to treat nausea, migraine, intestinal constipation, and rheumatic pain. The first evidence of smoking as a recreational drug was reported in 400 B.C. by the Greek historian Herodotus. In 800 A.D., smoking became common in the Middle East and South Asia. Only in the 20th century did its recreational use emerge, and many countries classified it as an illicit drug [1].

Components

The most abundant phytocannabinoids in this plant are ∆9-tetrahydrocannabinol (∆9-THC) and cannabidiol (CBD), compounds with a similar chemical structure that allows them to bind to the same receptor in the brain but produce very different effects since THC has a psychoactive effect [5]. In contrast, CBD has anxiolytic and antipsychotic properties [1].

Legalization

Since the early 2000s, cannabis has been decriminalized and legalized in many US states for medical purposes and some (the District of Columbia and 15 US states, Alaska, Arizona, California, Colorado, Illinois, Maine, Massachusetts, Michigan, Montana, Nevada, New Jersey, Oregon, South Dakota, Vermont, and Washington [21]) even for recreational use [1,12]. A bill to decriminalize cannabis was recently passed in the House of Representatives [4]. However, despite its legalization at the state level, the federal government has not taken critical legislative measures [12].

Currently, under federal law, the possession and use of cannabis are still illegal. Scientists still have limited access to study it because the US Drug Enforcement Agency (DEA) classifies it as a Schedule I substance [12].

After changes in state legislation, there has been a notable increase in public access to a variety of cannabis-containing products in North America, with high-potency cannabis flowers, ≥20% tetrahydrocannabinol, and high-potency cannabis concentrates (≥60% THC) dominating the recreational cannabis market and is linked to addictive behavior [9-10,15,22].

Epidemiology

**Figure 2:**
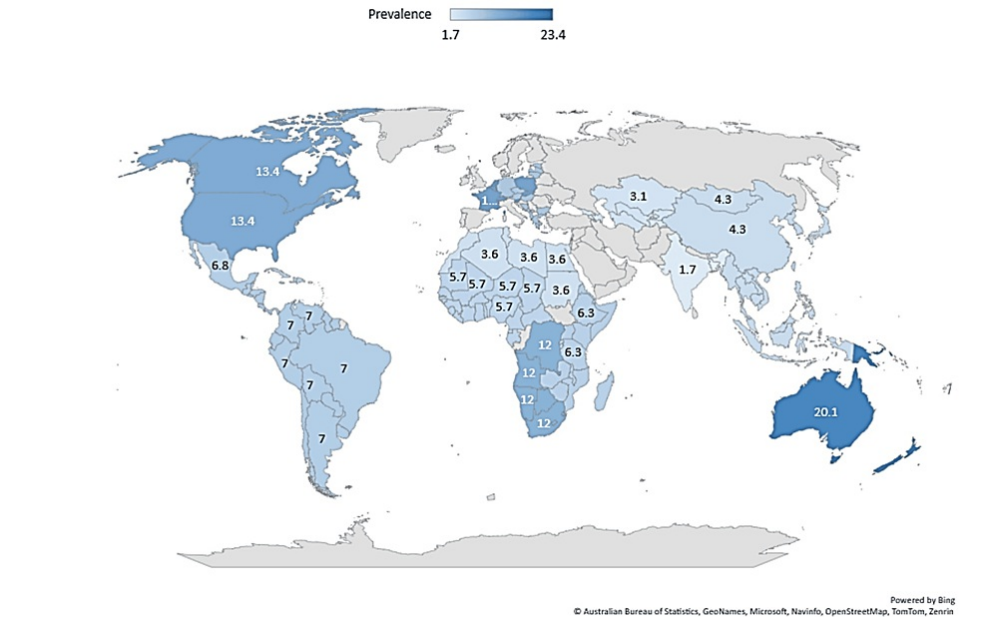
A blue-scale representation of the prevalence (percentage) of cannabis use in adolescents (aged 15-16) by country in 2023. Data was adapted from World Drug Report 2023, Statistical Annex Tables, Office on Drugs and Crime, United Nations Image Credit: MS

In 2020, cannabis was the most-used drug globally [23], with 209 million users, and the percentage of use in adolescence grows yearly [1]. Figure 2 shows the global prevalence of cannabis use among young people (aged 15-16) in 2023.

According to the United Nations, cannabis is the third most commonly used drug among young people aged 15-24, after tobacco and alcohol [1]. The prevalence of heavy daily use has tripled in the last 25 years, with 6.9% of US high school seniors reporting its usage. This represents an area of particular concern and creates a scientific, medical, and economic challenge due to changes in the political and social perception of cannabis [2,4-5], such as a reduction in the public perception of harm, greater accessibility, and changes in the types and modes of cannabis consumption [3,12,24].

In past decades, boys had higher consumption rates associated with the use of a greater variety of routes of administration (from smoking to oral and vaporization), products with high potency and concentration, compared with girls. However, the gap between men and women has been reduced recently, especially among adolescents. In addition, a "telescoping effect" has been identified in girls, demonstrating accelerated progression from first use to use disorder [2].

**Figure 3:**
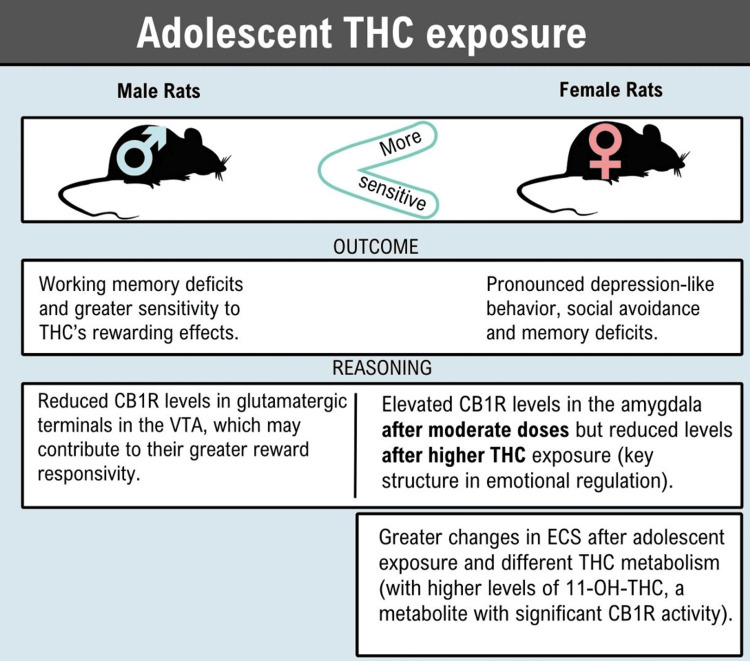
Outcomes found in experiments with rats and exposure to THC. Data was adapted from [11] Image Credit: MS THC: tetrahydrocannabinol; CB1R: cannabinoid receptor type 1; VTA: ventral tegmental area; ECS: endocannabinoid system; 11-OH-THC: 11-hydroxy-THC

Previous studies in rats show that female cannabis users are more susceptible to depression and males appear more prone to addiction, including a higher risk of developing a use disorder. Thus, a greater susceptibility to divergent psychiatric vulnerabilities in humans could be extrapolated [11]. Figure 3 shows the detailed findings of the study.

In general, consumption before the age of 16 has been associated with acute effects that cause a variety of negative mood states, such as tension, agitation, instability, paranoia, mental confusion, and memory impairment, and chronic effects such as increased susceptibility to substance use disorder and mental health, including personality disorders, anxiety, depression, cognitive impairment, disrupted brain maturation, and suicidality [2]. People with heavier use or CUD often have a lower quality of life [8-9], and it has been associated with poor educational performance, school dropout, and decreased satisfaction with life [5].

Main Components of Cannabis and Its Brain Impact

THC is a partial agonist of CB1 and CB2 receptors and has psychoactive properties with neurotropic effects that include "high" anxiety and psychosis, especially when consumed in high amounts [1,9], being classified as dangerous for this reason. On the other hand, CBD has been considered "safe" due to its ability to counteract the effects induced by THC. It acts as a negative allosteric modulator of both CBRs, reducing the potency and efficacy of CB1R agonists and acting as a partial agonist of dopamine two (D2) receptors. Each produces a different central effect, although they work on the endocannabinoid system (ECS), which are lipid neuromodulators that play a broad and critical role in numerous developmental processes to regulate synaptic transmission [11] in various physiological processes (e.g., motor control, pain perception, regulation of energy balance, and the immune system) [1].

Central effects of the main component of cannabis are shown in Table 8.

**Table 8:** Central effects of the main component of cannabis. THC: tetrahydrocannabinol; CBD: cannabidiol; DA: dopamine; PTSD: post-traumatic stress disorder

THC	CBD
It shows psychoactive properties and a rewarding effect (due to the induced dysregulation of mesolimbic DA transmission and affect salience stimuli evaluation) [1].	It doesn't show rewarding effects or psychoactive properties (due to the inability to alter extracellular DA levels in the ventral striatum). Still, it can normalize or restore aberrant DA signaling and salience processing [1].
During adolescence, neurodevelopmental trajectories change, leading to long-lasting effects (e.g., vulnerability to drug addiction and psychotic episodes) [1].	It represents a therapeutic strategy against different substance use disorders, reducing craving and withdrawal opioid symptoms [1].
May predispose to the abuse of other illicit drugs (e.g., cocaine, heroin, and amphetamines) in later adulthood, thus promoting drug dependence ("gateway hypothesis") [1].	Clinical studies have shown it can decrease social anxiety symptoms, sedation, anxiety, and cognitive impairment during speech performance as well as symptoms in PTSD patients [1].
From a neurobiological point of view, several preclinical studies have shown that it is neurotoxic to brain areas rich in type 1 cannabinoid receptors, including the hippocampus, the amygdala, the striatum, and the prefrontal cortex [15].	Brain imaging studies suggest that its anxiolytic effects could be due to its ability to decrease amygdala activation [1].

Cannabis and Youth

During adolescence, cannabis use is a particular concern, and research suggests that its use, especially early-onset, chronic, high doses, or potency [12], during mid-to-late adolescence, may be associated with altered cortical development, especially in CB1 receptor-rich prefrontal regions, which exhibit prolonged maturational trajectories [7,25].

Chronic CB1R agonist exposure throughout adolescence, a crucial time for neurodevelopment, can overstimulate the ECS, resulting in severe and enduring alterations that range from emotional and cognitive deficiencies to neuropsychiatric disorders [1]. It is essential to consider that the current presentations available on the market are high-potency strains with high THC concentrations and low CBD levels. According to some authors, it may be the reason for the increase in harmful effects associated with cannabis use [2], which can further influence brain development and cause worse results than previous generations of users [1].

**Figure 4:**
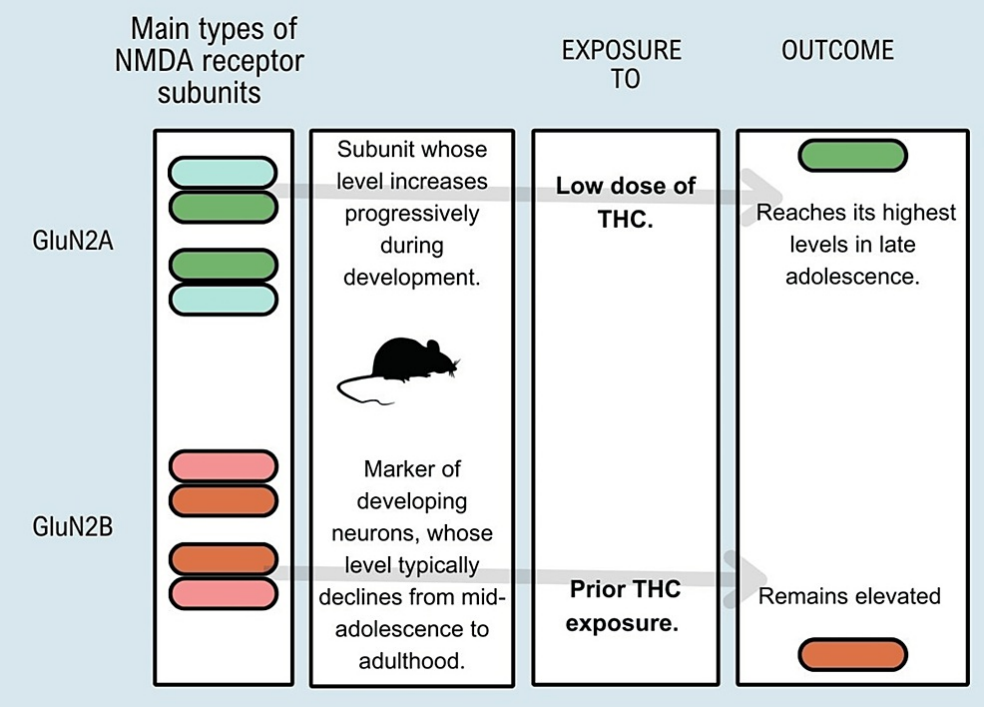
Outcomes found in experiments with rats and exposure to THC. Data was adapted from [11] Image Credit: MS NMDA: N-methyl-D-aspartate; TCH: tetrahydrocannabinol

THC exposure in adolescents increases overall levels of glutamatergic markers in adulthood. It alters the typical developmental trajectory of the expression of glutamate receptor subunits that have functional significance for synaptic plasticity, indicating premature maturation of crucial markers of plasticity, coinciding with the early pruning of dendritic spines, and simultaneously attenuating the average reduction in N-methyl-D-aspartate (NMDA) GluN2B receptor content, delaying different facets of normal development-representation in Figure 4 of findings found in experiments with rats and exposure to THC [11].

A critical, little-considered aspect of cannabis use is secondhand exposure during youth since studies have shown that young children exposed to secondhand cannabis smoke show emotional and cognitive problems. In the United States, cannabis use by parents with children in the home increased from 4.9% to 6.8% between 2002 and 2015. In addition, airborne particle monitoring studies in nearly 300 households of families with at least one child under the age of 14 showed that 15.1% had documented indoor cannabis smoking [11,26].

The evidence and association of secondhand cannabis smoke and its effects on development are minimal; however, considering studies that have documented the presence of significant concentrations of THC and its metabolites in oral fluids, blood, or urine samples from exposed children, it is essential to emphasize the need for more studies to determine short- and long-term neurobiological effects [11].

Neurodevelopmental Period During Adolescence

Brain development is a continuous process that begins early in the prenatal period through late adolescence and ends in early adulthood, where full maturity is reached [11]. It is especially in this last transition, characterized by rapid and sensitive changes [2]. Furthermore, synaptic pruning and increased myelination occur in long developmental trajectories of the frontal, parietal, and temporal regions. In particular, the prefrontal cortex, striatum, and amygdala, which exhibit synapse overproduction and regulate complex executive functions in humans [2,7], are especially susceptible to reward and affective processes. Throughout all stages of maturation, ECS has a fundamental regulatory role with functions such as signaling cell and neuronal migration, regulation of signaling pathways, and synaptic transmission in the CNS [11].

In this vital period of neurological maturation in adolescence, many youthful processes occur, including behavior setbacks and a considerable refinement of cognitive function, emotionality, and reward [7,11]. This will improve working memory, intelligence quotients, problem-solving, and specific behavioral phenotypes, such as risk-taking, sensation-seeking, and peer socialization, preparing the individual for adulthood [2].

An increased susceptibility to substance use accompanies these critical phases of substantial neuronal development [2,15,27]. According to results from human research, exposure to cannabis produces essential changes in the expected trajectory of cellular processing, neurocircuitry, and the ontogenetic profile of the ECS, which generate irreversible behavioral alterations later in life [11].

Cannabis use has been associated with interrupting the maturation of sustained attention and neurocognitive deficits in learning and memory. It has even been related to possible alterations in the brain macrostructure, causing atypical neuronal functioning that can cause lasting alterations [5,8,15]. Some studies indicate that long-term cognitive impairments occur primarily in early adolescent-onset (≤ age of 15 or 16) cannabis users [12].

Psychiatric and Personality Problems

According to the evidence, users with early onset [1,16], high frequency (≥ weekly), and high potency (≥10% Δ9-THC) have been demonstrated to have an impact on affective disorders, such as ideation and attempts at suicidal behavior (predominantly in people that show depression traits), and increase the risk of depression, bipolar disorder, partner violence, child maltreatment, motor vehicle collision, and rates of anxiety disorders including panic disorder, social anxiety disorder, and post-traumatic stress disorder [12,16,19].

Individuals with child maltreatment and specific genetic polymorphisms have shown a strong association between exposure to this drug and the later-life emergence of mental health disorder symptoms, suggesting that its interaction with the genotype increases the risk of conditions such as psychosis, schizophrenia, and bipolar disorder [2,5,12,16].

Cannabis users, compared to non-users, showed reduced sensitivity to loss-blunted responses to non-drug rewards, contributing to sensation-seeking, impulsivity, and, ultimately, addictive behaviors [9].

Neurologic and Cognitive Problems

Functional magnetic resonance imaging (fMRI) studies have shown changes in various brain regions during tasks in chronic users of cannabinoids, involving working memory [19], perceptual reasoning, processing speed [20], intelligence quotient, learning, cognitive functions [1,3,8], producing specific learning disorders, for instance, dyscalculia and dyslexia, executive control, reward processing, cannabis cue reactivity, and emotional processing, through the impact of CB1R and functional modulation of dopaminergic neuronal activation states [2-3,15].

Some experimental animal studies need to be proven in humans to demonstrate its effects, especially the TCH exposure during adolescence, which provokes neuroinflammatory phenotypes, increases brain cytokines [1], alters FOS protein expression in the CB1 receptor, impairs endocannabinoid-mediated synaptic plasticity, disrupts dendritic spine pruning, and reduces the numbers of prefrontal pyramidal neuronal spines in contrast with the CBD component, which has antioxidant and neuroinflammatory effects [1,5].

Statistical analysis showed that the chronic use of cannabis [18,28] significantly impacts the functioning of higher cognitive processes [15,29]. Also, it has been related to changes in cerebellar structure as it shares connections with dopaminergic systems in the basal ganglia and shows deficits in behavioral paradigms associated with this structure, such as eyeblink conditioning, disadvantageous decision-making [1], risk of mood, drug craving, anxiety disorders, psychotic symptoms, psychosis [3,11,14], and alteration of motivational processes [7,9].

**Figure 5:**
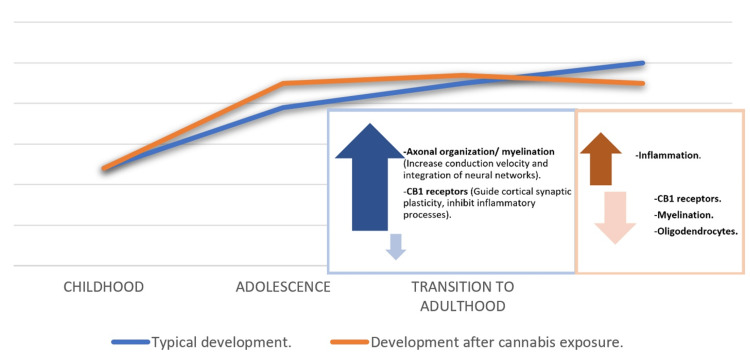
A theoretical model of developing anterior cingulate cortex and anterior thalamic radiations, connectivity among individuals with and without cannabis exposure (typical development, blue line, and development after cannabis exposure, orange line). Data was adapted from [13] Image Credit: MS CB1: cannabinoid receptor 1

It is thought that during adolescence, an essential target of cannabis on the brain is the white matter pathways, bundles of axons rich in myelin, specifically the cingulum and anterior thalamic radiations [13,30]. Even with minimum use, it reduces its maturation from ages 20 to 22. See Figure 5.

Changes in Brain Activation and Macrostructure in Cannabis Users

**Figure 6:**
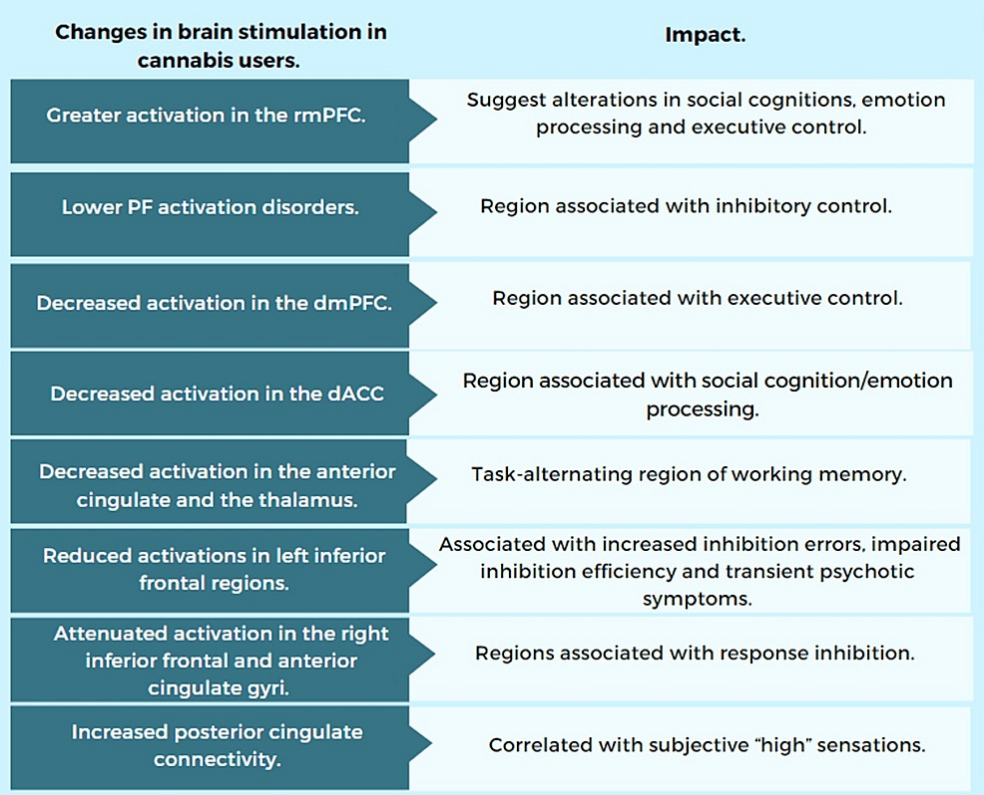
Changes in brain stimulation using fMRI in youth cannabis users. Data was adapted from [3] and [9] Image Credit: MS fMRI: functional magnetic resonance imaging; PF: prefrontal; PFC: prefrontal cortex; rm: rostral medial; dm: dorsal medial; dACC: dorsal anterior cingulate cortex

Multiple meta-regression analyses and meta-analyses differentiate areas of brain activation between users and non-users of cannabinoids. Youth CU showed the following findings, presented in Figure 6. Also, they presented macrostructural changes in the brain, such as reduced gray matter volume (GMV) in the lateral superior temporal gyrus (L-STG) and increased GMV in the right middle occipital gyrus (R-MOG) [17], decreased volume and surface area, altered thickness across frontal and parietal areas, smaller thalamic volume and larger amygdala volume changes in the dendritic architecture (premature pruning of spines and atrophy of arbors) [4,7,20], changes in the volume and shape of the hippocampus, and more vigorous growth and changes in cerebellar thickness in lobule VI and Crus l [4,14-15].

## Conclusions

The increased use and availability of cannabis worldwide, especially among the young population after its partial legalization, the new presentations available on the market (strains with high THC concentrations and low CBD), and their perception of its low harmfulness among the population should be a reason for concern among the scientific and health community. Since, according to previous research, mostly carried out on animals, its use at the level of the CNS is related to supposed adverse effects on cognitive and neurobehavioral development, this is what raises the following question: Is there a difference in neurocognitive impairments between children and adolescents who are exposed to cannabis and cannabinoid-containing substances (including vaping) and those who are not?

Based on the articles reviewed, its exposure, especially in vulnerable periods such as adolescence, could predispose to psychiatric vulnerabilities in humans, such as depression, anxiety, memory deficits, social avoidance, and addiction, and be the gateway to trying other drugs. Its use has been related to macrostructural changes in the brain and alterations in the activation of different brain areas when performing specific tasks. Given this reality, studies like this are essential to make visible this global problem that could cause permanent neurological changes in adolescents and eventually affect society. Today, there are few investigations carried out in humans with contradictory results, which have been limited by cross-sectional study designs, the participation of adult populations, small samples, and challenges in analyzing their short- and long-term effects. Once the main effect and impact on the CNS are known, suggestions could be made for the prevention and early intervention of cannabis disorders and recommendations for their regulation.
